# Supplementary material for: A general-purpose machine-learning force field for bulk and nanostructured phosphorus
Source: Nat Commun. 2020 Oct 29;11:5461. doi: 10.1038/s41467-020-19168-z (PMC7596484; doi:10.1038/s41467-020-19168-z)
Supplement: Supplementary file 1 — Supplementary Information [file 41467_2020_19168_MOESM1_ESM.pdf]

Supplementary Information for

A general-purpose machine-learning force field for bulk and nanostructured phosphorus

Volker L. Deringer,[1,*] Miguel A. Caro[2,3] & Gábor Csányi[4]

[1] *Department of Chemistry, Inorganic Chemistry Laboratory, University of Oxford, Oxford OX1 3QR, United Kingdom*

[2] *Department of Electrical Engineering and Automation, Aalto University, Espoo, 02150, Finland*

[3] *Department of Applied Physics, Aalto University, Espoo, 02150, Finland*

[4] *Engineering Laboratory, University of Cambridge, Cambridge CB2 1PZ, United Kingdom*

* E-mail: volker.deringer@chem.ox.ac.uk

Supplementary Note 1: Composition of the reference database

Here, we provide mroe details about the composition of the reference database (as a supplement to Figure 1 in the main text). The database consists of the following components:

A GAP-RSS dataset. The aim of these input data is to achieve diverse sampling of structural space; rather than very high accuracy: the objective is to achieve at least reasonable quality for any conceivable atomistic configuration, as might be encountered in a random search. There are still some limits to avoid the sampling of highly unreasonable configurations, *e.g.*, imposed by the use of a hard-sphere radius, which is a central strategy in *Ab Initio* Random Structure Searching (AIRSS)[1] and adapted in GAP-RSS;[2] similarly, symmetry operations are used to narrow down the number of required search attempts.[1] Structures for the present dataset are taken from ref. 3 and have been obtained there in iterative structure searches. Energies and forces for all these structures are re-computed here using PBE+MBD. The dataset includes (very) high energy randomised as well as progressively relaxed structures. In the final GAP-RSS iteration, only those structures were added which contained exclusively threefold-bonded atoms.[3] The regularisation is chosen to reflect this: generally, the data are fitted more "tightly" the more relaxed the structures are (Supplementary Table 1).

Liquid structures. Initially, we generated randomised configurations based on ensembles of P_4 molecules (using the `buildcell` code of the AIRSS suite[1]), and we performed short DFT-MD simulations of liquid structures at different densities using CP2K.[4] Then, iterative MD runs with interim versions of the GAP were performed, and single-point snapshots from these trajectories were added to the reference database, conceptually similar to prior work.[5,6] In the final potential, the initial configurations were discarded and all liquid reference configurations stem from GAP-MD. These simulations include both low- and high-density liquid P (molecular and network structures); some snapshots contain fragments relevant to both phases, which is thought to be useful for studies of the transition between both (*cf.* Figure 7).

2D structures. This is primarily phosphorene,[7] derived from black P, with some additional configurations added that correspond to As-type (A7) derived sheets[8] – the latter have also been discussed as possible monolayer materials (and are sometimes referred to as "blue P / blue phosphorene").[9] The structures were created by increasing the interlayer spacing of the underlying crystal structure by various amounts and randomising the atomic positions in the cells, while keeping the connectivity of the 2D extended layers intact. These are therefore relatively small cells, collectively labelled "Exfoliation" in Supplementary Table 1. Further structures were created by generating supercell models of larger sheets, or by cutting 1D extended ribbons from phosphorene, in both cases including the randomised removal of atoms to create point defects and the running of short GAP-MD simulations to sample thermally distorted structures.

Bulk crystals. We generated randomly scaled and distorted unit cells of the most important allotropes that are stable at ambient pressure (black,[10] white,[11] Hittorf's,[12] fibrous[13]) and high pressure (As-type, simple cubic), up to about 100 GPa.[8] We note that even more complicated forms occur at ultrahigh pressure (≫ 100 GPa), such as the incommensurately modulated P-IV structure.[14] We performed initial tests but decided to not include those phases in the final potential, and therefore define its high-pressure application range as up to and including simple cubic P.

Isolated fragments. We included dimer configurations (P_2) at spacings between 1.6 and 3.0 Å, aiming to describe both compression and stretching of the covalent bond, uniformly scaled P_4 tetrahedra with P–P distances between 1.9 and 3.5 Å, and configurations corresponding to the $P_4 \rightleftharpoons 2\ P_2$ dissociation. We stress that these are only included for robustness, not in an aim to describe all details of the molecular PES – which is expected to require even higher levels of theory. We finally include a free atom in a large supercell, which serves as energy reference in the fit.

Supplementary Table 1 | Composition of the reference database to which the model is fitted, and regularisation parameters for energies (σ_E), forces (σ_F), and virial stresses (σ_V) in the respective parts.

		Database Size		Regularisation		
		Cells	Atoms	σ_E (eV at.$^{-1}$)	σ_F (eV Å^{-1})	σ_V (eV)
GAP-RSS[3]	Initial (random)	199	1920	0.03	0.4	0.5
	Intermediates	995	9320	0.02	0.3	0.4
	Relaxed	596	5706	0.01	0.2	0.2
	3-coordinated	400	7412	0.005	0.1	0.1
Liquid	Network	164	40672	0.002	0.2	0.2
	Molecular (P_4)	88	21824	0.002	0.2	0.2
2D	Ribbons	40	4216	0.002	0.2	0.2
	Large sheets	87	11535	0.002	0.2	0.2
	Exfoliation	1234	14172	0.001	0.03	0.05
Bulk crystals		959	24292	0.0003	0.03	0.05
P_2 / P_4 molecules		35	110	0.002	0.2	0.2
Free atom		1	1	0.0001	0.01	–
**Total**		**4798**	**141180**			

More detailed information about the reference database can be gathered by colour-coding the points contained in the map in Figure 1. In Supplementary Figure 1, we indicate the (average) coordination number, *N*, per structure, similar in style to ref. 2. We separate the plot according to GAP-RSS (that is, essentially random-based) and manually created parts of the database; accordingly, the former is more uniformly distributed in the map; the latter shows the distinct "islands" which had already been seen in Figure 1 in the main text. Most structures represented in the central region of Supplementary Figure 1 (a) have an average coordination number of $N = 3$ (*green*), the coordination number in all known ambient-pressure allotropes of P, although there are some scattered low-coordinated structures (*blue*) and a region of large coordination numbers in the lower right part of the map (*yellow*).

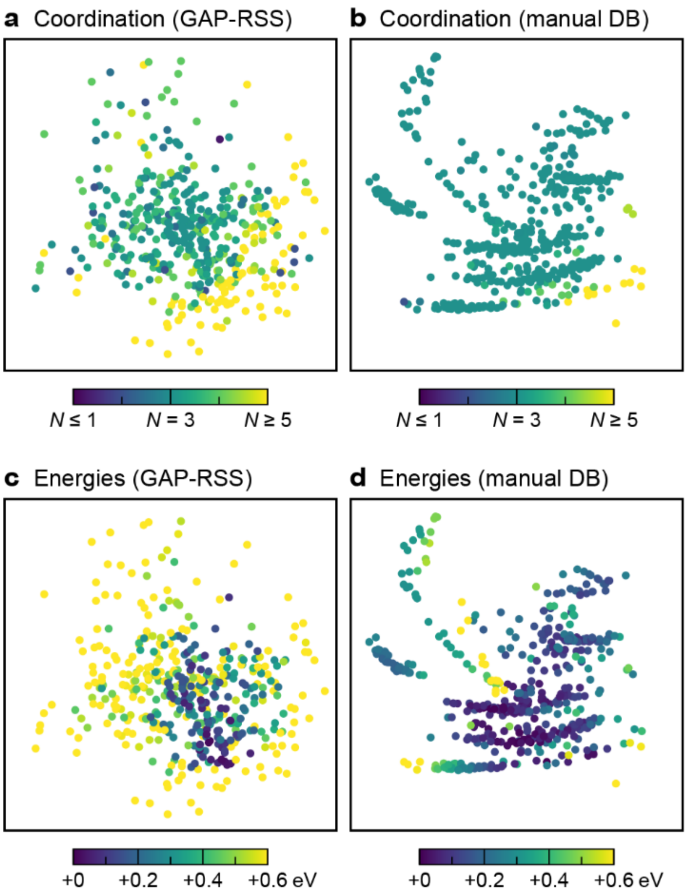

Supplementary Figure 1 | Composition of the reference database visualised in a 2D map, as in Figure 1, but now color-coded according to (**a**–**b**) coordination numbers, N, and (**c**–**d**) energy per atom above the minimum. Panels **a** and **c** show markers only for the GAP-RSS derived part of the database ("DB"), whereas panels **b** and **d** show those for the manually constructed part of it.

The vast majority of the manually created structures has $N = 3$ (panel b), although there is some atom-to-atom fluctuation in the dense liquid which is not seen in the per-cell averaged values which are used for colour-coding here. There is a small region of compressed structures with higher coordination numbers, again at the bottom right of the map.

It is also instructive to inspect the relative energy of different configurations in the reference database, which we do in panels c–d. As expected, the GAP-RSS dataset contains a number of higher-energy structures, although it reaches more favourable values in the central region of the map where the crystalline allotropes are found (Figure 1) – one may think of this

as the GAP-RSS iteratively "circling in" on stable structures,[2,15] and indeed we have previously reported how a GAP-RSS run easily discovered the black P structure within a few iterations.[3] The entries of the manually constructed part of the database (panel d) exhibit lower energies overall, with the only notable exceptions being regions of unusual coordination numbers. The distorted white P structures (located in the "island" on the left) have excess energies of about 0.2 eV, in line with the known metastability of this allotrope;[16] the energies for the molecular liquid phase are even somewhat higher. Similarly, the phosphorene ribbons (top right region of the map) have higher energies than the stable bulk and monolayer structures from which they are derived.

Supplementary Note 2: Custom regularisation

Supplementary Table 2 | Root mean square error (RMSE) measures, as in Table 1 in the main text (reproduced here in boldface), but now additionally for variants of the force field with different types of regularisation. Values in the header are given for (energies, forces, virials), in units of (eV at.$^{-1}$, eV Å^{-1}, eV), respectively.

		Loose regularisation throughout (0.03, 0.4, 0.5)		**Custom regularisation (Supplementary Table 1)**		Tight regularisation throughout (0.0003, 0.03, 0.05)	
		RMSE Energies (eV at.$^{-1}$)	RMSE Forces (eV Å^{-1})	**RMSE Energies** (eV at.$^{-1}$)	**RMSE Forces** (eV Å^{-1})	RMSE Energies (eV at.$^{-1}$)	RMSE Forces (eV Å^{-1})
GAP-RSS	Initial (rnd.)	0.089	0.70	**0.116**	**0.69**	0.092	0.67
	Intermed.	0.058	0.36	**0.055**	**0.38**	0.065	0.40
	Relaxed	0.055	0.33	**0.058**	**0.36**	0.041	0.33
	3-coord.	0.034	0.27	**0.032**	**0.26**	0.033	0.30
Network liquid		0.015	0.33	**0.008**	**0.36**	0.008	0.39
Molecular liquid		0.006	0.12	**0.002**	**0.15**	0.002	0.18
2D structures		0.013	0.12	**0.002**	**0.07**	0.004	0.11
Bulk crystals		0.006	0.16	**0.001**	**0.06**	0.002	0.14

Here, we assess the utility of a custom regularisation of the input data that is employed in the GAP framework. We fitted two other versions of our force field: one in which the expected errors for *all* the input data were set to the maximum value of all considered (that for the initial GAP-RSS configurations in the custom approach; "loose" regularisation), and one where they were set to a much smaller value (that for the crystalline phases in the custom approach; "tight") throughout. All other parameters of the fits were the same. The RMSE errors (Supplementary Table 2) provide a quality measure for the resulting force fields. Note the clearly improved description of the 2D structures and the bulk crystals when the custom regularisation is used (boldface), compared to *either* of the versions with constant regularisation throughout.

Supplementary Note 3: Black phosphorus

Supplementary Table 3 | Crystallographic data of black phosphorus (space group *Cmce*, no. 64, $Z = 8$, P on 8*f* with $x = ½$ and y and z as free parameters), comparing experimental reports with DFT+MBD and GAP+R6.

	(ref.)	*a* (Å)	*b* (Å)	*c* (Å)	*y*	*z*
Powder XRD,[a] 1935	(17)	3.31	10.50	4.38	0.098	0.090
Powder / SC-XRD, 1965	(10)	3.3136(5)	10.478(1)	4.3763(5)	0.10168(9)	0.08056(28)
Powder / SC-XRD, 2007	(18)	3.3164(5)	10.484(1)	4.3793(5)	0.10162(2)	0.08068(5)
Powder XRD, 2014	(19)	3.3264(2)	10.5194(8)	4.3937(2)	0.0970(4)	0.0871(4)
DFT+MBD (This work)		3.314	10.490	4.333	0.10246	0.07862
GAP+R6 (This work)		3.314	10.442	4.332	0.10317	0.07860

[a] Initially reported in *Bmab*; here transformed to the equivalent *Cmce* setting for easier comparison.

Here, we benchmark the optimised lattice parameters in the bulk against experimental references (Supplementary Table 3). We include in our survey the first report based on powder X-ray diffraction (XRD) from 1935,[17] as well as a foundational study combining powder and single-crystal (SC) XRD from 1965.[10] In such studies, the powder measurement is used for the lattice parameters and SC-XRD for the refinement of the atomic positions, using the most accurate approach in each case. We include two more recent experimental studies. One (2007) is based on a new synthesis route via the ternary phase Au_3SnP_7, leading to high-quality crystals;[18] this work largely confirms the 1965 results but offers substantially improved accuracy for the positional parameters. We finally include powder XRD results from a 2014 study, which similarly offers improved crystal quality over the much earlier work.[19]

Supplementary References

1. Pickard, C. J. & Needs, R. J. *Ab initio* random structure searching. *J. Phys.: Condens. Matter* **23**, 053201 (2011).

2. Bernstein, N., Csányi, G. & Deringer, V. L. De novo exploration and self-guided learning of potential-energy surfaces. *npj Comput. Mater.* **5**, 99 (2019).

3. Deringer, V. L., Proserpio, D. M., Csányi, G. & Pickard, C. J. Data-driven learning and prediction of inorganic crystal structures. *Faraday Discuss.* **211**, 45–59 (2018).

4. Kühne, T. D. *et al.* CP2K: An electronic structure and molecular dynamics software package - Quickstep: Efficient and accurate electronic structure calculations. *J. Chem. Phys.* **152**, 194103 (2020).

5. Deringer, V. L. & Csányi, G. Machine learning based interatomic potential for amorphous carbon. *Phys. Rev. B* **95**, 094203 (2017).

6. Sosso, G. C., Miceli, G., Caravati, S., Behler, J. & Bernasconi, M. Neural network interatomic potential for the phase change material GeTe. *Phys. Rev. B* **85**, 174103 (2012).

7. Liu, H. *et al.* Phosphorene: An Unexplored 2D Semiconductor with a High Hole Mobility. *ACS Nano* **8**, 4033–4041 (2014).

8. Jamieson, J. C. Crystal Structures Adopted by Black Phosphorus at High Pressures. *Science* **139**, 1291–1292 (1963).

9. Zhu, Z. & Tománek, D. Semiconducting Layered Blue Phosphorus: A Computational Study. *Phys. Rev. Lett.* **112**, 176802 (2014).

10. Brown, A. & Rundqvist, S. Refinement of the crystal structure of black phosphorus. *Acta Cryst* **19**, 684–685 (1965).

11. Simon, A., Borrmann, H. & Horakh, J. On the Polymorphism of White Phosphorus. *Chem. Ber.* **130**, 1235–1240 (1997).

12. Thurn, H. & Krebs, H. Über Struktur und Eigenschaften der Halbmetalle. XXII. Die Kristallstruktur des Hittorfschen Phosphors [in German]. *Acta Crystallogr., Sect. B* **25**, 125–135 (1969).

13. Ruck, M. *et al.* Fibrous Red Phosphorus. *Angew. Chem. Int. Ed.* **44**, 7616–7619 (2005).

14. Marqués, M. *et al.* Origin of incommensurate modulations in the high-pressure phosphorus IV phase. *Phys. Rev. B* **78**, 054120 (2008).

15. Deringer, V. L., Pickard, C. J. & Csányi, G. Data-Driven Learning of Total and Local Energies in Elemental Boron. *Phys. Rev. Lett.* **120**, 156001 (2018).

16. O'hare, P. A. G., Lewis, B. M. & Shirotani, I. Thermodynamic stability of orthorhombic black phosphorus. *Thermochim. Acta* **129**, 57–62 (1988).

17. Hultgren, R., Gingrich, N. S. & Warren, B. E. The Atomic Distribution in Red and Black Phosphorus and the Crystal Structure of Black Phosphorus. *J. Chem. Phys.* **3**, 351–355 (1935).

18. Lange, S., Schmidt, P. & Nilges, T. Au_3SnP_7@Black Phosphorus: An Easy Access to Black Phosphorus. *Inorg. Chem.* **46**, 4028–4035 (2007).

19. Köpf, M. *et al.* Access and in situ growth of phosphorene-precursor black phosphorus. *J. Cryst. Growth* **405**, 6–10 (2014).
